# Thermal Stability and Fire Properties of Salen and Metallosalens as Fire Retardants in Thermoplastic Polyurethane (TPU)

**DOI:** 10.3390/ma10060665

**Published:** 2017-06-17

**Authors:** Aditya Ramgobin, Gaëlle Fontaine, Christophe Penverne, Serge Bourbigot

**Affiliations:** 1 UMR 8207—UMET—Unité Matériaux et Transformations, University of Lille, Ecole Nationale Supérieure de Chimie Lille, F-59652 Villeneuve d’Ascq, France; aa.ramgobin@ed.univ-lille1.fr (A.R.); gaelle.fontaine@ensc-lille.fr (G.F.); 2 USR 3290—MSAP—Miniaturisation pour la Synthèse, l’Analyse et la Protéomique, University of Lille, F-59652 Villeneuve d’Ascq, France; christophe.penverne@univ-lille1.fr

**Keywords:** salen, metallosalen, thermoplastic polyurethane, fire retardants, PCFC, thermogravimetric analysis, Mass Loss Cone Calorimetry

## Abstract

This study deals with the synthesis and evaluation of salen based derivatives as fire retardants in thermoplastic polyurethane. Salens, hydroxysalens and their first row transition metal complexes (salen-M) were synthesized (Copper, Manganese, Nickel and Zinc). They were then incorporated in thermoplastic polyurethane (TPU) with a loading as low as 10:1 weight ratio. The thermal stability as well as the fire properties of the formulations were evaluated. Thermogravimetric analysis (TGA) showed that different coordination metals on the salen could induce different decomposition pathways when mixed with TPU. The Pyrolysis Combustion Flow Calorimetry (PCFC) results showed that some M-salen have the ability to significantly decrease the peak heat release rate (−61% compared to neat TPU) and total heat released (−63% compared to neat TPU) when formulated at 10:1 wt % ratio in TPU. Mass Loss Cone Calorimetry (MLC) results have shown that some additives (salen-Cu and salen-Mn) exhibit very promising performance and they are good candidates as flame-retardants for TPU.

## 1. Introduction

Flame-retardants (FR) are a wide range of substances that are used as additives in a plethora of polymeric materials with the aim of inhibiting, suppressing or delaying ignition in order to prevent the spread of fire [1]. The market today consists of a large variety of FR to contribute to general safety in many applications. 

The largest selling inorganic flame retardant by weight is presently aluminum tri-hydroxide (ATH). It is used as a filler in a wide range of elastomers, thermoplastics, and thermosetting resins processed at low temperatures (<200 °C). ATH requires a high loading (40–60 wt %) in order to meet acceptable fire properties. The addition of promoters in the polymeric systems to decrease filler proportions is one of the sought out routes to increase the efficiency of such FRs [2]. On the other hand, the most effective, generally applicable commercial fire-retardant systems presently available are based on halogen containing compounds. However, concerns regarding halogenated FR have caused a decline in the demand in the FR market. As a result, halogen free alternatives are being sought in this field [3]. Another type of FR that has gained popularity over the past decades is phosphorus based ones. Indeed, FR research as well as the market on phosphorus-based FR have proliferated partly due to the aforementioned increased demand in halogen-free FRs. However, despite the wide array of possibilities that can be exploited with such FRs, there are some issues regarding them. For instance, most inorganic phosphorus based FR additives require very high loadings (>30 wt %) in order to have a significant effect [4]. This high a loading can have adverse effects on the physical and mechanical properties of the material [1]. Moreover, because phosphorus-based FR may, in some cases, release phosphoric acid, corrosion-related issues of such additives arise [5].

In order to tackle the aforementioned issues, a new class of fire retardant showing promising preliminary results has been developed. The work performed at the laboratory deals with the study of different classes of FR. Indeed, with the aim of developing novel FRs with high efficiency and low toxicity, one such class of FR that was developed concerns *N*,*N*′-(bis salicylidene)ethylenediamine (salen, hereafter called **s1**) and *N*,*N*′-bis(4-hydroxysalicylidene)ethylenediamine (salen(OH)_2_, hereafter called **s2**) as well as their copper (II) complexes. 

Salens have been extensively studied in different fields, namely, as a catalyst in asymmetric synthesis [6]. Metallosalens have shown to be effective catalysts, which induced the cleavage of DNA [7]. Salens and hydroxysalens have also shown promise in the field of enantioselective epoxidation [8].

They have proved to exhibit highly interesting fire properties in thermoplastic polyurethane (TPU) [9]. Previous studies have shown that **s1** has the potential of acting as FR in TPU with loadings as low as 10 wt % [10]. Previous studies on salens have thoroughly investigated the decomposition mechanism of **s2** [11]. It was demonstrated that, under high temperature conditions, **s2** has the ability to undergo a polycondensation reaction and crosslink, forming a thermally stable residue; however, **s1** did not exhibit this behavior [10]. For this reason, despite the fact that they both exhibit interesting fire properties, it is believed that **s1** and **s2** have different modes of fire retardation. In addition, the complexation of **s2** with copper revealed that degradation temperature of copper–salen complex increases. Indeed, the presence of copper ion as a metallosalen additive in TPU has been shown to enhance the fire properties of TPU/salen formulation. The fire retardant mechanism is reported in an unpublished work where the salen–Cu complex has been shown to work synergistically with the thermal degradation of TPU. It has been reported that metal ions can coordinate to a fragmented section of TPU, keeping it in a condensed phase, and action of the radical formed is also shown [12].

In the pursuit of our effort for developing new FRs, and, in particular, salen-based FRs, the effect of the metallic cation complexed in salens was investigated. Indeed, the choice of the cation should influence the degradation mechanism of the FR as well as its fire properties and provides a promising route to designing new FR. Salen had previously been used in thermoplastic polyurethane (TPU). Therefore, the focus of this work concerns the screening of metallosalen (salen metal complexes, M-salen) in order to determine their thermal stability and investigate their efficiency as FRs in the same polymer. Should an enhanced stability with similar fire properties arise, these FRs would have the potential for wider applicability in polymeric materials with higher processing temperatures.

## 2. Results and Discussion

### 2.1. Synthesis and Characterization

#### 2.1.1. Salen Ligand

**Figure 1:**
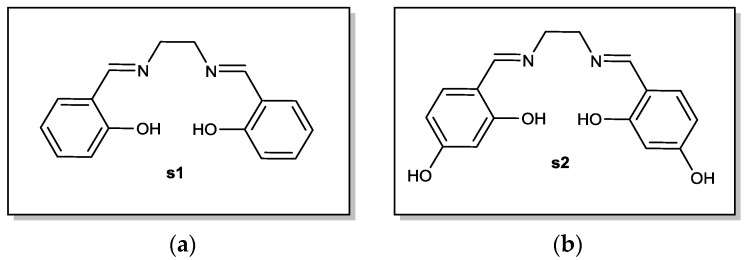
Structures of (**a**) the base salen ligand **s1** and (**b**) and salen(OH)_2_, **s2**.

The salen ligand **s1** (Figure 1a) and salen(OH)_2_ **s2** (Figure 1b), which are the products of the condensation reaction between the corresponding salicylaldehyde and ethylenediamine, were synthesized using a very simple procedure previously described [9]. The reaction yields were particularly high with 94% for **s1** and 96% for **s2**.

The ligands were characterized by nuclear magnetic resonance (NMR) spectroscopy (^1^H, ^13^C and heteronuclear coupling) as well as Infra Red (IR) spectroscopy. 

#### 2.1.2. Salen Complexes

**Figure 2:**
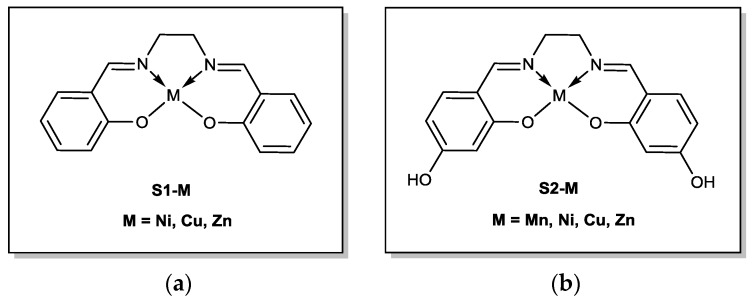
(**a**) Panel of metallosalen (labelled **s1-M**); (**b**) different metallosalen(OH)_2_ (labelled **s2-M**).

A range of salen and salen(OH)_2_ complexes (Figure 2) were synthesized. Synthetic methods had to be adapted with respect to the sensitivity of the reactants that were used. The metals that were used were limited to first row earth abundant metals [13] and zinc. Most of the complexes afforded a relatively acceptable yield ranging from 40% to 80%. However, due to its high air sensitivity, **s1-Mn** was not synthesized. 

Most of the complexes were characterized by NMR techniques (^1^H and ^13^C). However, some of the metal ions that were used for the complexation reaction were highly paramagnetic, making their identification via conventional NMR pulse programs impossible. Therefore, IR spectroscopy and mass spectroscopy were also performed to characterize them.

### 2.2. Thermal Stability

#### 2.2.1. Thermogravimetric Analysis of **s1**, **s2** and Their Complexes

**Figure 3:**
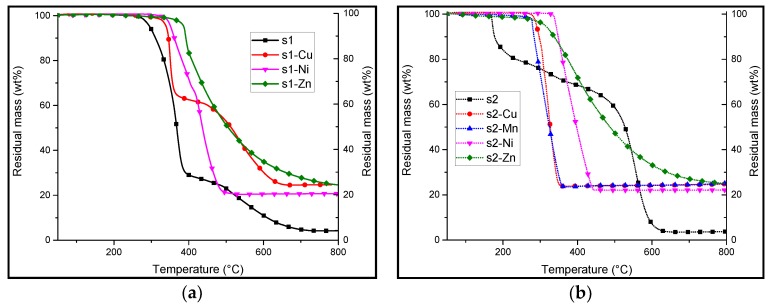
Thermogravimetric analyses (TGA) at a heating rate of 10 °C/min in air of salen (**a**) black: **s1**; salen complexes: red: **s1-Cu**; magenta: **s1-Ni** and dark green: **s1-Zn** and (**b**) salen(OH)_2_: gray: **s2**, salen(OH)_2_ complexes: orange: **s2-Cu**; lime: **s2-Mn**; indigo: **s2-Ni**, violet: **s2-Zn**.

**Figure 4:**
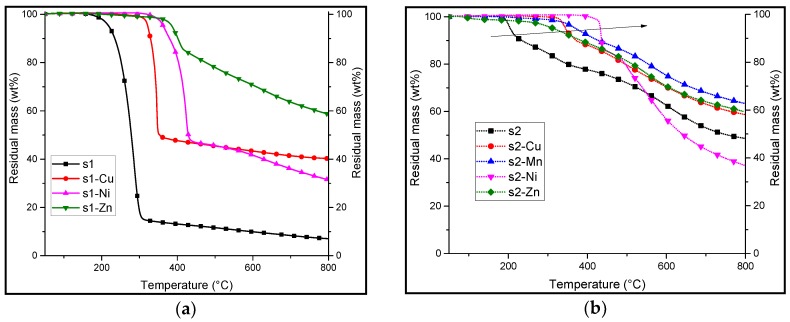
Thermogravimetric analyses (TGA) at a heating rate of 10 °C/min under nitrogen of (**a**) salen **s1** (black) and salen complexes: **s1-Cu** (red), **s1-Ni** (magenta), **s1-Zn** (green) and (**b**) salen(OH)_2_, **s2** (black), salen(OH)_2_ complexes: **s2-Cu** (red), **s2-Mn** (blue), **s2-Ni** (magenta) and **s2-Zn** (green). The black arrow on figure 4b shows the increase in thermal stability of the complexes.

The thermo-oxidative and pyrolytic decomposition of salens **s1** and **s2** and their corresponding complexes were studied (Figure 3 and Figure 4, Table 1 and Table 2). It can be observed that the presence of a coordination metal greatly influences the thermal stability of the substance whatever the atmosphere (nitrogen or in air). A significant increase in the degradation temperature is observed when comparing the thermogravimetric analysis (TGA) plots of the different complexes to the beginning of the decomposition temperature of the base salens **s1** and **s2**. Indeed, while the TG plot shows that **s1** starts to degrade at 295 °C, those of the complexes show a decomposition temperature that is at least 45 °C higher (in the case of **s1-Cu**). Similarly, in the case of **s2** (decomposition temperature of 171 °C), the TGA plots of **s2** complexes show that they start decomposing at a temperature that is at least 108 °C higher. This enhanced thermal stability may be explained by the strong interaction between the metal ion and the chelating ligand with the oxygen donor atoms. The residual mass at 800 °C are also significantly higher when the salens are chelated with a metal.

In air, **s1** undergoes a sharp first degradation step at 295 °C with a mass loss corresponding to about 65% of the initial mass (at 400 °C). After this step, there is a slow decrease in residual mass until a stable final residue is reached at about 700 °C. This behavior may be attributed to the formation of a transient char that is relatively stable, which undergoes slow decomposition compared to the rate of initial degradation. 

Similar thermal behaviors are observed when **s1** is complexed with the different metals (data summarized in Table 1). The presence of a coordination metal chelated to the salen ligand increases its thermal stability. Indeed, the initial degradation temperatures increase significantly when there is a metal attached to the salen framework. In the case of **s1**-**Zn**, the initial degradation temperature is as high as 386 °C (+91 °C as compared to that for **s1**). In terms of the first degradation step, the TGA curve of **s1**-**Cu** shows a sharp mass loss at 340 °C, which is shortly followed by another decomposition step at around 400 °C. This behavior seems to be similar to the decomposition of **s1**, albeit occurring at a higher temperature. 

However, **s1**-**Zn** undergoes slow, continuous degradation starting at about 400 °C, suggesting that there is the formation of a transient char right after the start of the decomposition. This is observed during the mass loss cone calorimetry (MLC) test (discussed later), whereby the char formation is observed. **s1-Ni** decomposes with two successive steps occurring at around 367 °C (corresponding to a mass loss of 34 wt %) and 423 °C (with a mass loss of 46%). 

In the case of **s2** and **s2-M**, initial degradation occurs at lower temperatures than does that for **s1** and **s1-M**. The thermal decomposition of **s2** occurs in two major steps similar to that for **s1**. The first step corresponds to a lower mass loss and takes place at a lower temperature (170 °C). 

From previous studies of the thermal degradation of **s2**, it is known that **s2** has the ability to form a phenolic resin. This occurs via a polycondensation reaction involving the formation of methylene bridges sandwiched between aromatic rings. It was also shown that water is evolved at around 190 and 200 °C. This suggests that there is some form of condensation reaction occurring. This could explain the relatively low temperature degradation of **s2** (170 °C) that corresponds to degradation of **s2**, which forms fragments that subsequently react to form a transient char, which is assumed to be a thermally stable polycondensed phenolic resin [10]. The degradation of this char (556 °C) occurs at a higher temperature than does **s1** (500 °C). 

On the other hand, the TGA plots of **s2-M** complexes, except that for **s2-Zn**, show a single sharp decomposition at around 300–400 °C, followed by the formation of a highly stable residue. This is attributed to the metal oxide of the cation used. **s2-Zn** has a different decomposition behavior as compared to the other **s2-M** complexes. Indeed, as for **s1-Zn**, a slow, continuous decomposition of the complex as early as at the start of the degradation is observed in the TGA plot. Despite the residual mass being relatively close to that for the other complexes, it suggests that there is a different mode of decomposition with zinc.

Different decomposition behaviors are observed when the TGA are carried out under pyrolysis conditions (Table 2). Indeed, **s1** degrades firstly with a sharp step that begins at 219 °C (*T*_5%_). After the first degradation, there is a slow, linear decrease in residual mass (from 15 wt % at 309 °C to 7 wt % at 800 °C).

Similar results are observed when the **s1** is complexed with the different metal cations where the decomposition of the **s1-M** complexes occur in a single step. **s1-Cu** degrades at 322 °C and leaves a residual mass of 40 wt %. It has the lowest degradation temperature among the complexes. **s1-Ni** decomposes at a higher temperature (363 °C as compared to **s1-Cu**) with a lower residual mass (31 wt %), and **s1-Zn** has the highest thermal stability with a decomposition temperature of 383 °C. It also has the highest remaining mass of 58 wt % at 800 °C.

As it was expected from the above discussion, the degradation temperatures depend on the coordination metal used (degradation temperature increased from 290 to 386 °C in the case of **s1-Zn**). Moreover, there is a significantly higher residual mass compared to the neat ligand at the end of the measurement (at 800 °C), strengthening the hypothesis that the metal complex causes the formation of a more stable char. Most samples have a relatively high residual mass at 800 °C (*M*_res_ > 30 wt %).

In the case of **s2** under pyrolytic conditions, the decomposition also starts at a lower temperature than **s1**. However, it occurs at a much lower rate and corresponds to a lower mass loss (about 10 wt % instead of about 85 wt %). It is followed by a slow degradation with respect to temperature, yielding a residue of around 49 wt % (about seven times more than **s1**). 

Similar behavior is observed by most salen(OH)_2_ complexes, **s2-M**. Their TGA plots show a sharp mass loss at 350–400 °C followed by slow degradation, except for **s2-Mn**, with which the TGA plot shows only a continuous, slow rate of degradation. This suggests that there might be a different mode of degradation with these complexes as compared to the other **s2** complexes. 

Table 3 illustrates the calculated weight percent of the metal oxides with the assumption that the decomposition of the complex led only to the formation of the metal oxides. Under inert atmosphere, the remaining mass at 800 °C is significantly higher than the calculated weight percent of the metal oxide. This suggests that there might be the formation of a thermally stable structure. The general trend suggests that the **s2-M** complexes have a slightly higher residual mass than the **s1-M** complexes.

This enhancement of the thermal stability was already reported [11]. The **s2** ligand showed the ability to form a stable cross-linked phenolic resin under thermal stress. In this study, it has been shown that the presence of a metal in the **s2** ligand keeps the FR’s ability to form a stable material at high temperatures. The chelation by different metals increases the degradation temperature of the FR, thus, extending their applicability to polymers with higher processing temperatures.

The results show that the salen and metallosalen complexes would remain stable at the processing temperature of TPU (190 °C). 

#### 2.2.2. Thermogravimetric Analysis (TGA) of TPU/Salen Formulations

**Figure 5:**
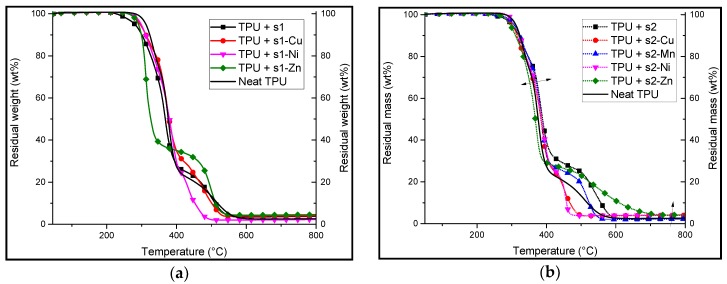
TGA analyses under thermo-oxidative conditions (air) of TPU formulations with 10:1 wt % ratio of (**a**) **s1** and its corresponding complexes; (**b**) **s2** and its corresponding complexes. The double ended arrow shows the small variation in the decomposition temperatures of the formulations.

**Figure 6:**
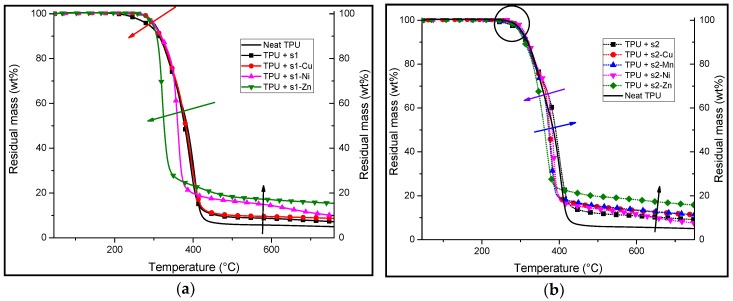
TGA analyses under inert atmosphere (N_2_) of TPU formulations with 10:1 wt % ratio of (**a**) **s1** and its corresponding complexes; (**b**) **s2** and its corresponding complexes. The red arrow shows the general trend of the decomposition temperature of the additives. The green arrow shows the relatively low temperature at which the **s1-Zn** decomposes as compared to the rest of the formulations. The black arrows show increased residual masses at the second stage of the decomposition. The violet and blue arrows show the increase or decrease in the mass loss rates. The circle shows the initial decomposition stage of the formulations.

TPU formulations containing 10:1 weight ratio of TPU/additives (salen and their complexes) were subjected to TGA analyses in nitrogen (Figure 5) and in air (Figure 6). The aim was to evaluate the effect (stabilization or destabilization) of the FRs compared to the neat polymer (Table 4 and Table 5).

In thermo-oxidative atmosphere, the TGA curves of all formulations including neat TPU show a two-step decomposition process. All of the metal complexes based formulations show a much lower decomposition temperature at 5 wt % ranging from 290 to 298 °C as compared to neat TPU (312 °C). 

The second decomposition step corresponds to the degradation of a transient char, which decomposes at a higher temperature than the first decomposition temperature. In the case of TPU/**s1-Zn**, the TGA curve shows a first step that occurs with a very high mass loss rate (2.3 wt %/s as compared to 1.5 wt %/s for neat TPU) and a second step occurring at a much higher temperature than the others (450 °C). Based on previously reported work, it suggests that there is formation of a stable compound after the first step of the decomposition. This compound remains relatively stable at high temperatures. Such a behavior is known to occur with the dihydroxy(salen) when it is subjected to a high temperature stress. Indeed, dihydroxysalen has the ability to form cross-linked polymeric structures when subjected to thermal stress [11]. With the exception of TPU/**s1-Ni**, there is a slight increase in residual mass of the formulations.

With the exception of zinc-based formulations, the TGA curves of TPU/**s2** formulations show similar behaviors as TPU/**s1** formulations under air. The TGA plots of all the formulations exhibit a two-stage decomposition. The first degradation temperatures range from 295 to 311 °C, with TPU/**s2-Zn** starting its degradation at the lowest (295 °C) temperature and that of **s2-Ni** at the highest (311 °C). The mass loss rates (MLR) are also relatively close to the MLR of neat TPU. However, in the case of **s2**, **s2-Mn** and **s2-Zn**, the TGA plots show that the second degradation of the formulations occurs at a higher temperature than the other formulations (>500 °C). It is assigned to the ability of **s2** to form thermally stable resins at high temperatures [9]. The presence of these metal ions in the complex may catalyze this mechanism of resin formation and polycondensation making a greater amount of the thermally stable, cross-linked structure [11]. 

In pyrolytic conditions (Figure 6 and Table 5), TPU/**s1** shows a slight decrease of the thermal stability compared to that of neat TPU and TPU/**s1-M** complexes. Indeed, the decomposition of the TPU/**s1** starts at 287 °C whereas that of neat TPU and the other formulations show an initial mass loss (95 wt %) at a higher temperature of around 300 °C. 

However, despite showing a higher decomposition temperature than TPU/**s1**, the TGA plots of both TPU/**s1-Ni** and TPU/**s1-Zn** show that they have a first decomposition stage that is steeper than the other formulations (green arrow, Figure 6a). They also correspond to a lower mass loss than do the other **s1**-**M** formulations. Moreover, all of the formulations show a residual mass at 750 °C that is higher than that of the neat TPU (black arrow, Figure 6a). This suggests that there is polymer/FR interactions promoting charring.

TGA of TPU containing **s2** and its complexes show that they have similar thermal behavior to neat TPU in terms of the first decomposition temperature under nitrogen (encircled), which begins around 300 °C for every formulation. Moreover, there is a higher residual mass at 750 °C for the formulated samples (black arrow, Figure 6b) as compared to that of neat TPU. The TGA curve of TPU/**s2-Zn** shows the highest remaining mass at 750 °C (15 wt %). It is noteworthy that the mass of the corresponding metal oxide does not account for the totality of the residual mass. The TGA curves of **s2-Cu** and **s2-Ni** show higher mass loss rates (MLR) than the other formulations (Table 5). 

However, the TGA curve of TPU/**s2-Zn** shows an apparent single-step decomposition unlike the other formulations, which appear to decompose in two steps, as for neat TPU. The apparent single-step decomposition of TPU/**s2-Zn** is assigned to the overlapping of the two decomposition stages of the formulation. Indeed, this is made evident on the DTG (derivative thermogravimetric) curve, whereby two distinct steps are observed (curve not shown). The residual masses at 750 °C are significantly higher for all the **s2**-complex formulations compared to that of neat TPU (as high as 50 wt % more in the case of TPU/**s2-Zn**). This suggests that there is the formation of thermally robust char.

In conclusion, it is observed that 10:1 wt % ratio of salen or their complexes in TPU can significantly change the thermal stability of the TPU/salen system. The results suggest increased charring and have shown different mass loss rates (MLRs). This suggests that the mode of decomposition of TPU may be altered simply by mixing it with a small amount (10:1 wt % ratio) of salen or metal–salen complex.

**Figure 7:**
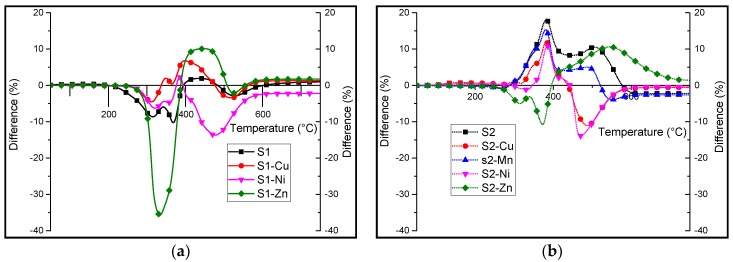
Differential thermogravimetric analysis (experimental percentage residual mass—calculated percentage residual mass) under thermo-oxidative atmosphere of (**a**) **s1** and the complexes and (**b**) metallosalen complexes when formulated with TPU at 10:1 wt % ratio.

Differential TGA under air is shown in Figure 7. This was undertaken to point out the differences in stabilization/destabilizations brought about by the presence of the different additives. Regarding TPU/**s1** and **s1-M** formulations, a major destabilization is observed in the differential TGA curve. This destabilization is much higher in the case of TPU/**s1-Zn**, whereby the curve peaks at a value as high as 35% between 300 and 400 °C. It is followed by a stabilization of around 10% at high temperatures (400–500 °C). However, with the exception of the TPU/**s1-Ni** formulation, there is mostly a stabilization of the system in this temperature range. In the case of **s1-Ni**, a destabilization is observed for the whole range of temperatures with a most important one between 400 and 600 °C. TPU/**s2** and **s2-M** formulations behave differently. Indeed, with the exception of TPU/**s2-Zn**, there appears to be a stabilization of 10–18% at about 380 °C in the differential TGA plot. However, the curve corresponding to TPU/**s2-Zn** shows a destabilization between 280 and 400 °C followed by a stabilization of 10% between 400 and 600 °C. It suggests that the interactions could stabilize char at high temperature, and so it should bring benefits in terms of reaction to fire. 

### 2.3. Pyrolysis Combustion Flow Calorimetry

Pyrolysis combustion flow calorimetry (PCFC) was carried out on both the flame-retardants and the formulated samples. This evaluation provides us with results regarding the specific heat release rate (HRR), the temperature at which the peak heat release rate (pHRR) occurs as well as the total heat released. The results are summarized in Table 6. 

**Figure 8:**
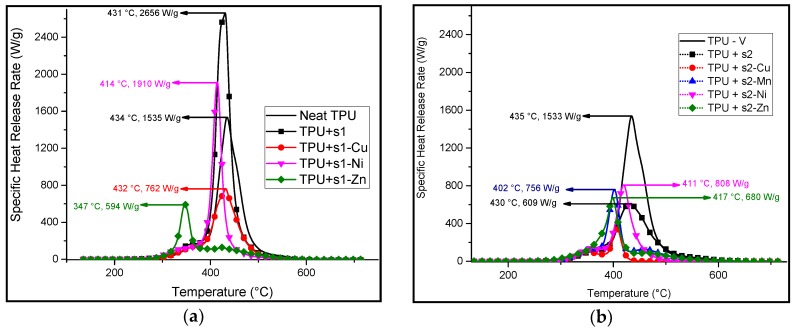
Corrected pyrolysis combustion flow calorimetry (PCFC) curves of (**a**) neat TPU, TPU/**s1** (10:1 wt % ratio) and TPU/**s1-metal** (10:1 wt % ratio); (**b**) neat TPU, TPU/**s2** (10:1 wt % ratio) and TPU/**s2-metal** (10:1 wt % ratio). The values of the temperature at the peak heat release rate (pHRR) as well as the pHRR are shown by the arrows.

Apart from **s1** and **s1-Ni**, the PCFC curves of every TPU containing **s1**-based additives show a decrease in the pHRR compared to that of neat TPU. The **s1-Zn** formulation has the lowest temperature at pHRR (Figure 8a) and it has the lowest total heat released (HR) (Table 6). For the other **s1**-complexes, the pHRR occurs between 410 and 435 °C. The peaks exhibit different widths at half height and have only one maximum. This broadness implies that the combustibles in the tested samples are released at a wide range of temperatures.

In the case of the formulations containing **s2** and its complexes, the PCFC curves of all the samples show a decrease in the pHRR (Figure 8b) and total heat released (Table 6) compared to neat TPU. The lowest pHRR is observed on the PCFC curve of TPU/**s1-Cu** (680 W/g), corresponding to a decrease of 56% as compared to that of neat TPU. The lowest total heat released (THR) corresponds to the TPU/**s2-Mn** formulation with 38 kJ/g compared to 90 kJ/g for neat TPU (−58%). It appears that additional peaks are observed before and after the pHRR. This multi-peak phenomenon may correlate to the TGA curves of the TPU/**s2** and TPU/**s2-M** (Figure 6b). The two-step decomposition of the TPU/**s2** formulations may thus be further inferred from the PCFC results above.

It is worth noting that there is, on average, more than a 50% decrease in the total heat release rate when the TPU/**s2** complexes are tested with TPU in the PCFC. 

**Figure 9:**
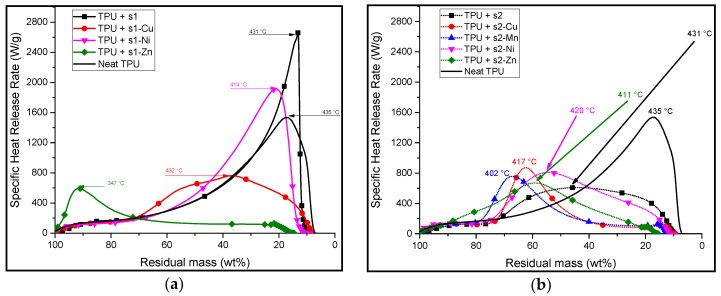
HRR vs. mass loss curves of (**a**) **s1** and its corresponding complexes and (**b**) **s2** and its corresponding complexes.

In order to investigate further the decomposition behavior of the formulations, the PCFC results were correlated to the TGA curves i.e., the specific heat release rate as a function of residual mass. This allowed observing the evolution of the mass of the sample as well as the degree of decomposition at which there is a notable release of combustibles. This led to a representation of the amount of heat released with respect to the remaining mass of the samples (Figure 9). The same scales were used for both sets of curves for comparison purposes. 

Except for that of TPU/**s1-Zn**, the HRR/mass-loss curves of TPU/**s1** formulations (Figure 9a) show an increase in HRR starting at around 60% of residual mass. The HRR/mass-loss curve of the formulation containing TPU and **s1** (black square, Figure 9a) has a much higher peak HRR than neat TPU. This suggests that most of the heat is released towards the end of the degradation of the samples and that there is a greater amount of combustibles released in TPU/**s1** compared to neat TPU. The HRR/mass-loss curve of TPU/**s1-Ni** (magenta down-pointing triangle, Figure 9a) sample shows a slightly broader peak than that of neat TPU. However, the overall shape of the curve is very similar to the neat TPU curve, suggesting that the degradation of these formulations is somewhat similar in terms of HRR. The HRR/mass-loss curve of TPU/**s1-Cu** (red) shows a much broader range over which HRR is relatively low. This suggests that the pyrolysis of the TPU/**s1-Cu** releases combustible materials over a wider range of its mass during its degradation, but also at a smaller rate. The curve corresponding to TPU/**s1-Zn** shows a very different shape when compared to the others. Most of the heat is released at the beginning of its decomposition. After that, there is a relatively slow HRR until about 15% residual mass.

The curves corresponding to TPU/**s2-M** (Figure 9b) formulations show varied results. The HRR/mass-loss curves of most of the TPU/**s2-M** show that they start to significantly release heat at around 85–80 wt %. However, it is interesting to note that there seems to be a preliminary phase between 0 and 20% weight loss whereby there is a constant heat release rate of approximately 100–150 W/g. This suggests that the early decomposition products of these formulations release non-combustible or hardly combustible products at the beginning of the pyrolysis. In the case of neat TPU, there is a slow and continuous increase of the HRR until the peak HRR is reached when the remaining mass is 17 wt %. Once again, the curve corresponding to TPU/**s2-Cu** (red, filled circle, dotted) formulation shows a very broad peak with a relatively low peak HRR.

From the combination of HRR/Temperature and HRR/mass-loss curves, it can be observed that TPU/**s1-Cu** and TPU/**s2-Cu** release low-combustibility combustibles over a wide range of residual mass. 

The TPU/**s1-Zn** formulation exhibits an intriguing behavior in the sense that most of its combustibles are released at a very early stage of its pyrolysis. Moreover, it has shown a relatively low peak heat release rate as compared to other **s1**-based formulations.

Finally, formulations of TPU and salen derivatives have shown that it can bring about a decrease in the HRR of the formulations by modifying the degradation steps of the formulations. This means that there would be a lower amount of combustibles released in a fire scenario. This suggests that these additives may have some fire retardant properties when they are incorporated in TPU.

### 2.4. Fire Properties Mass Loss Cone Calorimetry (MLC) Test

**Figure 10:**
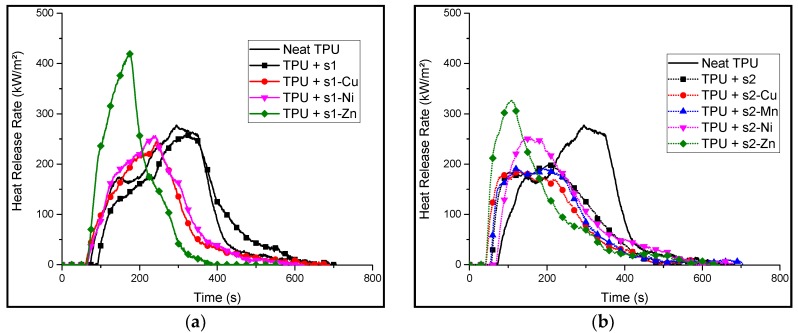
Mass loss cone calorimetry (MLC) curves of neat TPU, TPU/**s1-M** complexes (**a**) and TPU/**s2-M** complexes (**b**) on samples (50 × 50 × 3 mm^3^) exposed to 35 kW/m^2^.

In order to investigate the reaction to fire of the formulations, a mass loss cone calorimetry (MLC) test with a heat flux of 35 kW/m^2^ was performed on the formulated samples to simulate a fire scenario. The HRR curves are presented on Figure 10. 

The samples show similar behaviors right after ignition. There is a relatively steep increase in HRR at the beginning. A swelling of the sample is also observed in most cases. This swelling leads to the formation of a carbonaceous layer (char) with different thermal and mechanical robustness (visual observation and manual testing). Indeed, in the case of a thermally and mechanically stable char, the later can slow down the decomposition of the polymeric material lying under it. This can eventually limit the release inflammable gases, causing a lower HRR [14].

With the exception of Zn based formulations, the HRR curves of all the samples showed a decrease in the pHRR compared to neat TPU. The data are summarized in Table 7.

**Figure 11:**
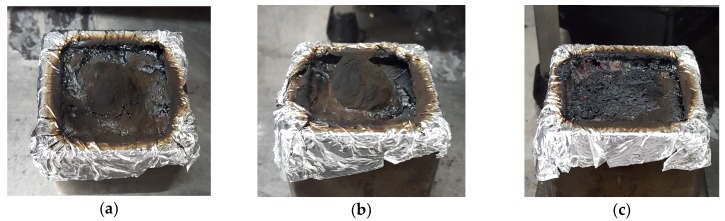
Residues of neat TPU (**a**); TPU/**s1** (**b**) and TPU/**s1-Cu** (**c**).

The HRR plot of neat TPU (solid line) shows two pHRR and a total heat released of 66.7 MJ/m^2^. It shows two HRR peaks. The first step starts at the ignition whereby there is a steep increase in HRR. This increase peaks at around 176 kW/m^2^ and 147 s. After this, a slight decrease in HRR is observed until around 175 s (277 kW/m^2^). This is assigned to the formation of a char that protects the unburnt layer of the material and delays its decomposition. However, at around 200 s, another sharp increase in the HRR is observed. This suggests that the inflammable decomposition gases are eventually released despite the formation of the char. The peak HRR is reached at this step (at around 300 s) and corresponds to a heat release rate of 277 kW/m^2^. The resulting residue is shown on Figure 11.

In the case of TPU/**s1**, the HRR curve shows no significant change brought about in the HRR values, neither in terms of pHRR nor in terms of THR. 

TPU/**s1-Cu** and TPU/**s1-Ni** behave differently as compared to neat TPU and TPU/**s1**. HRR curves exhibit only one peak occurring before that of neat TPU (250 s vs. 350 s) but the value of pHRR is not significantly decreased compared to neat TPU (−1.8%). A potential explanation for this behavior is that the char has a relatively low thermomechanical strength, causing it to break or not form at all. This can be observed in the photo of TPU/**s1-Cu** (Figure 11c). However, these formulations have a significantly lower total heat released (THR) as compared to neat TPU (−27.3% for Cu and −22.2% for Ni). 

**Figure 12:**
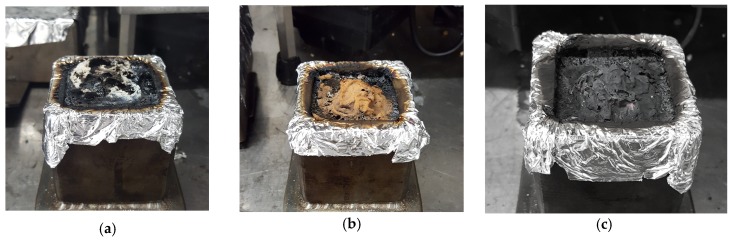
Samples of TPU/**s1-Zn** (**a**), TPU/**s2-Mn** (**b**) and TPU/**s2-Cu** (**c**) after the MLC test.

MLC results for TPU/**s1-Zn** formulation showed a very high pHRR (420 kW/m^2^) as compared to neat TPU (+51%). This may correlate to the TGA of TPU/**s1-Zn**, which showed a very drastic decrease in mass at a relatively low temperature (290 °C), therefore causing the rapid release of combustibles early on during its decomposition under the radiative flux. A relatively sharp peak is observed right after ignition. It suggests that the sample degraded very quickly, releasing a lot of heat, and then forms a protective layer, which limited the release of energy. Evidence of the char formation of TPU/**s1-Zn** can be seen in the picture taken after the MLC test (Figure 12). However, the char being relatively fragile, it did not keep its shape and collapses. The high pHRR is assigned to the possibility that the presence of zinc catalyzed the degradation process of the formulation (as seen in the TGA), causing a rapid burning of the sample. 

Formulations of **s2** and its complexes exhibit a lower pHRR and THR as compared to its **s1** counterparts. Indeed, the HRR curve of TPU/**s2** itself shows a pHRR of 200 kW/m^2^, which is about 28% lower than that of neat TPU and 23% lower than that of TPU/**s1**. This could be correlated to the fact that **s2** has the ability to form thermally stable cross-linked charred structure (polyphenol) at high temperatures.

The HRR as a function of a time curve of neat TPU, **s2** and **s2-M** formulations are relatively similar immediately after ignition. There is the formation of a char, which inhibits the decomposition of the sample. In the case of **s2** and **s2-M** formulations, this char is maintained. This results in the plateau that is observed in the HRR curves of TPU/**s2-Cu** and TPU/**s2-Mn**. This also explains the lower pHRR of these samples (pHRR < 200 kW/m^2^, as compared to neat TPU with a pHRR at 277 kW/m^2^). Indeed, the shapes of the HRR curves of these two formulations are consistent with HRR curves of materials that exhibit charring when they are burnt [14]. These observations suggest that the cross-linking ability of **s2** may have been maintained even if the ligand is complexed with Cu^2+^ or Mn^2+^. 

As with TPU/**s1-Zn**, the HRR curve of TPU/**s2-Zn** shows a higher pHRR compared to neat TPU (+17.7%). However, the **s2-Zn** formulation induces a lower pHRR than TPU/**s1-Zn**. This may be due to the ability of **s2** to form a cross-linked polyphenolic structure in the polymer matrix, forming a protective thermally stable layer, thus decreasing the pHRR. The zinc cation may catalyze the formation of the cross-linked structure. These bond formations being exothermic, it could explain the exceedingly high pHRR as compared to neat TPU.

The HRR curves of TPU/**s2-Cu** and TPU/**s2-Mn** show the lowest pHRR and THR of all of the formulations. The values are similiar and are consistent with PCFC results. Indeed, when compared to neat TPU, they showed a relatively low pHRR both in the PCFC test (−56% for Cu and −51% for Mn as compared to neat TPU) and in the MLC test (−32.5% for Cu and −30.7% for Mn). These results have shown that there is no distinct correlation between MLC and PCFC results. Indeed, considering that after the PCFC tests, the most promising sample as a fire retardant was the TPU/**s1-Zn** formulation. However, upon performing MLC, it was seen that, among all of the samples tested, TPU/**s1-Zn** has the highest pHRR, making it a mediocre candidate as a FR.

In conclusion, some of the formulated samples have shown that they exhibit enhanced reaction to fire evidenced by their lower pHRR as compared to neat TPU. On the other hand, other formulations have shown that they have a total heat released that is significantly lower than that of neat TPU. With the exception of TPU/**s1**, all of the formulations showed a THR, which was at least 15% lower than that of neat TPU. 

## 3. Materials and Methods

### 3.1. Instrumental Analyses

#### 3.1.1. Thermogravimetric Analysis

Thermogravimetric analyses (TGA) were conducted on a Setaram apparatus model TG 92-16 (Caluire-et-Cuire, France). Samples of 6–10 mg were placed in open alumina pans and heated either under nitrogen atmosphere or in air with a heating rate of 10 °C/min. 

#### 3.1.2. Pyrolysis Combustion Flow Calorimetry

Fire performance was evaluated based on heat release rate (HRR) curve obtained from an FAA Micro calorimeter (FAA Fire testing technology, East Grinstead, UK) operated at 1 °C/s to 750 °C in the pyrolysis zone according to ASTM D7309 method A. The combustion zone was set at 900 °C. Oxygen and nitrogen flow rates were set at 20 and 80 mL/min, respectively. Repeatability is confirmed by three consecutive trials (error margin ± 10%). 

The results obtained were corrected after conducting a TGA under nitrogen atmosphere of each sample. The conditions of the TGA were the same as that of the PCFC (1 °C/s to 750 °C, under nitrogen atmosphere). The residual mass at a given temperature allowed the calculation of the specific heat release rate at any given temperature.

#### 3.1.3. Mass Loss Cone Calorimeter

The mass loss cone calorimeter (Fire testing technology (FTT), Fire testing technology, East Grinstead, UK) is used for recording HRR curve. Plates (50 × 50 × 3 mm^3^ plates) for cone calorimeter test were made via compression molding using a Darragon press apparatus (Pinette P.E.I, Chalon sur Saône, France). Plates were wrapped in aluminum foil leaving the upper surface exposed to the heater and placed in a horizontal position on a ceramic block encased in a metallic container at a distance of 35 mm from the cone base. An external heat flux of 35 kW/m^2^ was used for all of the experiments. 

### 3.2. Synthesis and Characterisation

Salen and hydroxysalens **s1** and **s2** were obtained using a conventional procedure for salen synthesis based on the condensation of salicylaldehyde or its hydroxy-derivatives with diamine [6] Most salen complexes were synthesized according to procedure described in [5]. Zinc–salen complexes were prepared following the procedure described in [8].

Reagents were purchased from Aldrich (Sigma-Aldrich Chimie S.a.r.l., Lyon, France) or Lancaster Synthesis (Alfa Aesar, Haverhill, MA, USA) and were used without further purification. Diethyl ether, methanol and absolute ethanol were reagent grade commercial solvents and were used without further purification.

NMR spectra were recorded on a Bruker Avance 300 spectrometer (Billerica, MA, USA). Chemical shifts (δ) are referenced to internal solvent and given in ppm. Coupling constants (*J*) are given in Hz. The following abbreviations apply to spin multiplicity: s (singlet), d (doublet), t (triplet), q (quartet), m (multiplet) and bs (broad singlet).

#### 3.2.1. *N*,*N*′-Bis(salicylidene)ethylenediamine (**s1**)

To a solution of ethylenediamine (5.34 mL; 4.8 g; 0.08 mol) in 60 mL of absolute ethanol, salicylaldehyde (16.7 mL; 19.5 g; 0.16 mol) in 80 mL of ethanol was added dropwise with vigorous stirring. The product precipitated immediately and the mixture was refluxed for two hours, and then kept at room temperature and filtered. The product recrystallized from EtOH was filtered, washed with cold ethanol and ethyl ether, and then dried at 80 °C to give the desired Schiff base **s1** as yellow crystals, 20.2 g (94%). ^1^H-NMR (DMSO-*d*_6_) δ 3.92 (s, 4 H), 6.81–6.95 (m, 4 H), 7.35 (m, 4 H), 8.57 (s, 2 H); ^13^C-NMR (DMSO-*d*_6_) δ 58.8 (CH_2_), 116.4 (C_Ar_), 118.5 (C_Ar_), 118.6 (C_Ar_), 131.6 (C_Ar_), 132.3 (C_Ar_), 160.4 (C_Ar_), 166.8 (CN). MS (NanoESI) *m*/*z* 269.1 (M + H)^+^. Mp = 130 °C.

#### 3.2.2. *N*,*N*′-Bis(4-hydroxysalicylidene)ethylenediamine (**s2**)

According to the method described for **s1**, 21.9 g (0.16 mol) of 2,4-dihydroxybenzaldehyde and 5.34 mL (4.8 g, 0.08 mol) of ethylenediamine yielded compound **s2** (28.1 g; 94%) orange solid; ^1^H-NMR (DMSO-*d*_6_) δ 3.77 (s, 4 H), 6.15 (d, *J* = 2.4 Hz, 2 H), 6.26 (dd, *J* = 2.4, 8.6 Hz, 2 H), 7.16 (d, *J* = 8.6 Hz, 2 H), 8.35 (s, 2 H); ^13^C-NMR (DMSO-*d*_6_) δ 57.8 (CH_2_), 102.5 (C_Ar_), 106.9 (C_Ar_), 111.2 (C_Ar_), 133.4 (C_Ar_), 161.7 (C_Ar_), 164.3 (C_Ar_), 165.7 (CN). MS (NanoESI) *m*/*z* 301.2 (M + H)^+^. Mp > 190 °C (decomposition).

#### 3.2.3. *N*,*N*′-Bis(salicylidene)ethylenediamine Copper (II) Complex, **s1-Cu**

*N*,*N*′-bis(salicylidene)ethylenediamine **s1** (8.1 g, 0.03 mol) was dissolved in 70 mL absolute ethanol; then, a solution of copper (II) acetate (6.0 g, 0.03 mol) in 40 mL of water was added dropwise and the mixture was refluxed under vigorous stirring for 2.5 h. The green precipitate was collected by filtration, washed thoroughly with ethanol and then dried at 80 °C to give *N*,*N*′-bis(salicylidene)ethylenediamine copper (II) complex **s1-Cu** (6.96 g, 70%) as a fine green powder. NMR spectra of the complex was not recorded due to paramagnetism of Cu(II) (this well-known phenomenon leads to poor resolution of the spectra). MS (NanoESI) *m*/*z* 330.0 (M + H)^+^, 659.1 (dimer + H)^+^. Mp > 260 °C.

#### 3.2.4. *N*,*N*′-Bis(salicylidene)ethylenediamine Nickel (II) Complex, **s1-Ni**

*N*,*N*′-bis(salicylidene)ethylenediamine **s1** (6.01 g, 0.022 mol) was dissolved in 70 mL absolute ethanol, then a solution of nickel (II) acetate (5.51 g, 0.022 mol) in 40 mL of water was added dropwise and the mixture was refluxed under vigorous stirring for 2.5 h. The orange precipitate was collected by filtration, washed thoroughly with ethanol, and then dried at 80 °C to give *N*,*N*′-bis(salicylidene)ethylenediamine Nickel (II) complex **s1-Ni** (5.87 g, 80.6%) as an orange powder. ^1^H-NMR (300 MHz, DMSO-*d*_6_) δ ppm 3.29–3.35 (m, 1 H) 3.42 (s, 4 H) 6.51 (t, *J* = 7.25 Hz, 2 H) 6.70 (d, *J* = 8.67 Hz, 2 H) 7.17 (ddd, *J* = 8.43, 6.92, 1.79 Hz, 2 H) 7.25 (dd, *J* = 7.82, 1.60 Hz, 2 H) 7.89 (s, 2 H). ^13^C-NMR (75 MHz, DMSO-*d*_6_) δ ppm 58.45 (s, 1 C) 114.75 (s, 1 C) 120.22 (s, 1 C) 120.79 (s, 1 C) 133.22 (s, 1 C) 133.98 (s, 1 C) 163.07 (s, 1 C) 164.40 (s, 1 C). MS (NanoESI) *m*/*z* 324.0 M^+^. Mp > 260 °C. 

#### 3.2.5. *N*,*N*′-Bis(salicylidene)ethylenediamine Zinc (II) Complex, **s1-Zn**

The complex **s1-Zn** was prepared by first treating salicylaldehyde (2.15 mL, 0.02 mol) with zinc (II) acetate (2.26 g, 0.01 mol) in methanol (100 mL) for 30 min at room temperature. Then, ethylenediamine (0.68 mL, 0.01 mol) was added to the solution, and stirring was maintained at room temperature overnight. During that time, a light yellow precipitate formed. The precipitate was collected by filtration, washed with cold methanol and ether, and dried at 80 °C, affording 3.00 g, 80%. ^1^H-NMR (300 MHz, DMSO-*d*_6_) δ ppm 3.72 (s, 4 H) 3.92 (s, 1 H) 6.34–6.50 (m, 2 H) 6.55–6.68 (m, 1 H) 6.61 (d, *J* = 8.29 Hz, 1 H) 7.09–7.44 (m, 4 H) 8.43 (s, 2 H) 13.34–13.45 (m, 1 H). ^13^C-NMR (75 MHz, DMSO-*d*_6_) δ ppm 55.75 (s, 1 C) 112.15 (s, 1 C) 119.31 (s, 1 C) 122.68 (s, 1 C) 132.77 (s, 1 C) 134.73 (s, 1 C) 167.97 (s, 1 C) 171.01 (s, 1 C). MS (NanoESI) *m*/*z* 331.0 (M + H)^+^. Mp > 260 °C.

#### 3.2.6. *N*,*N*′-Bis(4-hydroxysalicylidene)ethylenediamine Copper (II) Complex, **s2-Cu**

*N*,*N*′-bis(4-hydroxysalicylidene)ethylenediamine **s2** (9.0 g, 0.03 mol) was dissolved in 70 mL absolute ethanol, then a solution of copper (II) acetate (6.0 g, 0.03 mol) in 40 mL of water was added dropwise and the mixture was refluxed under vigorous stirring for 2.5 h. The purple precipitate was collected by filtration, washed thoroughly with ethanol, and then dried at 80 °C to give *N*,*N*′-bis(4-hydroxysalicylidene)ethylenediamine copper (II) complex **s1-Cu** (9.7 g, 89%) as a purple powder. NMR spectra of the complex was not recorded due to paramagnetism of Cu(II) (this well-known phenomenon leads to poor resolution of the spectra). MS (NanoESI) *m*/*z* 362.0 (M + H)^+^. Mp > 260 °C.

#### 3.2.7. *N*,*N*′-Bis(4-hydroxysalicylidene)ethylenediamine Manganese (II) Complex, **s2-Mn**

*N*,*N*′-bis(4-hydroxysalicylidene)ethylenediamine **s2** (3.60 g, 0.012 mol) was dissolved in 70 mL absolute ethanol, then a solution of manganese (II) acetate (2.94 g, 0.012 mol) in 40 mL of water was added dropwise and the mixture was refluxed under vigorous stirring for 2.5 h. The brown precipitate was collected by filtration, washed thoroughly with ethanol, and then dried at 80 °C to give *N*,*N*′-bis(4-hydroxysalicylidene)ethylenediamine manganese (II) complex **s1-Mn** (3.1 g, 73%) as a brown solid. NMR spectra of the complex was not recorded due to paramagnetism of Mn(II) (this well-known phenomenon leads to poor resolution of the spectra). MS (NanoESI) *m*/*z* 353.0 (M)^+^. Mp > 260 °C.

#### 3.2.8. *N*,*N*′-Bis(4-hydroxysalicylidene)ethylenediamine Manganese (II) Complex, **s2-Ni**

*N*,*N*′-bis(4-hydroxysalicylidene)ethylenediamine **s2** (7.00 g, 0.022 mol) was dissolved in 50 mL absolute ethanol, then a solution of nickel (II) acetate (5.51 g, 0.022 mol) in 50 mL of ethanol was added dropwise, and the mixture was refluxed under vigorous stirring for 2. The red precipitate was collected by filtration, washed thoroughly with ethanol, and then dried at 80 °C to give *N*,*N*′-bis(4-hydroxysalicylidene)ethylenediamine cobalt (II) complex **s2-Ni** (5.36 g, 89%) as a red powder. ^1^H-NMR (300 MHz, DMSO-*d*_6_) δ ppm 3.25–3.31 (m, 2 H) 6.04 (d, *J* = 8.69 Hz, 2 H) 6.05 (s, 1 H) 7.05 (d, *J* = 8.10 Hz, 1 H) 7.62 (s, 1 H) 9.73 (br s, 1 H). ^13^C-NMR (75 MHz, DMSO-*d*_6_) δ ppm 58 (s, 1 C) 104 (s, 1 C) 106 (s, 1 C) 115 (s, 1 C) 135 (s, 1 C) 161 (s, 1 C) 163 (s, 1 C) 166 (s, 1 C). MS (NanoESI) *m*/*z* 356.0 (M)^+^. Mp > 260 °C.

#### 3.2.9. *N*,*N*′-bis(4-hydroxysalicylidene)ethylenediamine Manganese (II) Complex, **s2-Zn**

The complex **s2-Zn** was prepared by first treating 4-hydroxysalicylaldehyde (2.82 g, 0.020 mol) with Zn(OAc)_2_·2H_2_O (2.26 g, 0.010 mol) in methanol (70 mL) for 30 min at room temperature. Then, ethylenediamine (0.68 mL, 0.01 mol) in 35 mL of methanol was added to the solution, and stirring was maintained at room temperature overnight. During that time, a yellow precipitate formed. The precipitate was collected by filtration, washed with cold methanol and ether, and dried under vacuum, affording 3.23 g, 87%. ^1^H-NMR (300 MHz, DMSO-*d*_6_) δ ppm 3.59 (s, 2 H) 5.89–5.96 (m, 1 H) 5.90–5.95 (m, 1 H) 5.95 (s, 1 H) 5.98 (s, 1 H) 6.92 (d, *J* = 8.48 Hz, 1 H) 8.21 (s, 1 H) 9.39 (br s, 1 H). ^13^C-NMR (75 MHz, DMSO-*d*_6_) δ ppm 56 (s, 2 C) 103 (1 C) 107 (1 C) 113 (1 C) 136 (1 C) 162 (1 C) 167 (1 C) 173 (1 C). MS (NanoESI) *m*/*z* 363.1 (M + H)^+^. Mp > 260 °C.

### 3.3. Formulations, Processing and Sampling of Polymer

Thermoplastic Polyurethane (TPU) C85A (polyester polyurethane) was kindly supplied by BASF (Ludwigshafen, Germany).

TPU is dried for 12 h at 70 °C before use. Compounding of formulations with a ratio of 10:1 wt % (TPU:additive) of salen and their complexes was performed using a Microextruder (DSM Micro15, Xplore Instruments BV, Sittard, The Netherlands) under a nitrogen atmosphere at 190 °C (50 rpm for 5 min of mixing). The formulation was ground in liquid nitrogen in an ultracentrifuge to produce a powder and dried at 80 °C for 6 h before use. 

## 4. Conclusions

This work was devoted to the synthesis of some metallosalen and metallosalen(OH)_2_ with the aim of testing them as new FR in thermoplastic polyurethane and comparing them with the copper–salen complex, which has already been studied before. Seven metallosalens were successfully synthesized using simple chemistry in one or two-step reactions using readily available low-impact reactants (ethanol, salen and earth abundant metals). These substances were easily incorporated in TPU. Relatively low loadings with 10:1 weight ratios of TPU/FR were used. These formulations have shown promise in the field of FR. Moreover, MLC results are promising in the sense that the total heat released by the formulations are significantly lower than that of neat TPU. PCFC results of the formulation have also shown promising results with a decrease in HRR as low as 50– in the case of TPU/**s2-Cu** or TPU/**s2-Mn** and a decrease in THR of 40–60%. This confirms that this class of flame-retardants has a lot of potential in the field of polymer chemistry. By extension, the fire properties of the TPU/metallosalen complexes can be explained by the cross-linking ability of **s2** under thermal stress. However, as the metallosalens have an enhanced thermal stability, their applicability may be extended to engineering polymers with higher processing temperatures. 

## Figures and Tables

**Table 1:** Thermogravimetric data of salen, and their complexes under thermo-oxidative atmosphere at 10 °C/min.

Metal Complex	s1					s2				
	*T*_5%_ (°C)	*T*_MAX1_ (°C)	*T*_MAX2_ (°C)	*MLR* (%/°C)	*Res* (%)	*T*_5%_ (°C)	*T*_MAX_ (°C)	*T*_MAX2_ (°C)	*MLR1* (%/°C)	*Res* (%)
-	295	369	-	1.5 ^1^	4	171	171 ^1^	556	1.3 ^1^	4
Cu	340	349	325	2.4 ^1^	25	290	285 ^1^	325	1.3 ^1^	23
Mn	N/A	N/A	N/A	N/A	N/A	279	279 ^1^	-	1.5 ^1^	25
Ni	351	428	368	0.9 ^2^	20	342	347 ^1^	-	0.9 ^1^	22
Zn	386	392	-	1.1 ^1^	25	313	386 ^1^	-	1.4 ^1^	25

^1^ MLR_Max_ occurs at the first step of the degradation; ^2^ MLR_MAX_ occurs at the second step of the degradation. MLR: Mass loss rate; Res: Residual mass.

**Table 2:** Thermogravimetric data for the decomposition of salen in an inert atmosphere at 10 °C/min.

Sample	s1					Sample	s2				
	*T*_5%_ (°C)	*T*_MAX_ (°C)	*T*_MAX2_	*MLR* (%/°C)	*Res* (%)		*T*_5%_ (°C)	*T*_MAX1_ (°C)	*T*_MAX2_ (°C)	*MLR* (%/°C)	*Res* (%)
-	219	287 ^1^	-	1.7 ^1^	7	-	206		200	0.1 ^1^	49
**s1-Cu**	322	328	345	4.8 ^2^	40	**s2-Cu**	345	334	345	0.26 ^2^	59
**s1-Mn**	N/A	N/A	N/A	N/A	N/A	**s2-Mn**	371	375	550	0.1^2^	63
**s1-Ni**	363	368	424	1.7 ^2^	31	**s2-Ni**	432	435	502	1.6^1^	37
**s1-Zn**	386	405 ^1^	-	0.4^1^	58	**s2-Zn**	314	330	555	0.1^2^	60

*T*_5%_ corresponds to the temperature at which a mass loss of 5 wt % is recorded. ^1^ MLR_Max_ occurs at the first step of the degradation; ^2^ MLR_MAX_ occurs at the second step of the degradation.

**Table 3:** wt % of metal oxides as compared to the actual remaining masses of the complexes after thermogravimetric analysis (TGA) in inert and thermo-oxidative atmosphere.

Sample	s1			s2		
	MO (%) ^1^	Res (%) (N_2_)	Res (%) (Air)	MO (%) ^1^	Res (%) (N_2_)	Res (%) (Air)
Cu	25	40	25	22	59	23
Mn	N/A	N/A	N/A	20	63	25
Ni	23	31	20	21	37	22
Zn	24	58	25	22	60	25

^1^ weight percent of metal oxides with respect to the complex. MO: Metal Oxide.

**Table 4:** Thermogravimetric data for TPU/salen or salen complexes (10:1 weight ratio) formulation in air at 10 °C/min.

Sample	s1					Sample	s2				
	*T*_5%_ (°C)	*T*_MAX1_ (°C)	*T*_MAX2_ (°C)	*MLR* (%/°C)	*Res* (%)		*T*_5%_ (°C)	*T*_MAX1_ (°C)	*T*_MAX2_ (°C)	*MLR* (%/°C)	*Res* (%)
Neat TPU	312	331	376	1.5 ^2^	3	Neat TPU	312	331	376 ^2^	1.5 ^2^	3
TPU/**s1**	280	367	505	1.2 ^1^	4	TPU/**s2**	303	335	384 ^2^	1.2 ^2^	2
TPU/**s1-Cu**	295	316	374	1.1 ^2^	4	TPU/**s2-Cu**	302	317	370 ^2^	1.2 ^2^	4
TPU/**s1-Mn**	N/A	N/A	N/A	N/A	N/A	TPU/**s2-Mn**	307	332	388 ^2^	1.3 ^2^	2
TPU/**s1-Ni**	298	328	391	1.1 ^2^	2	TPU/**s2-Ni**	311	332	378 ^2^	1.4 ^2^	4
TPU/**s1-Zn**	290	312	501	2.3 ^1^	5	TPU/**s2-Zn**	295	310	371 ^2^	1.4 ^2^	5

^1^ MLR_Max_ occurs at the first step of the degradation; ^2^ MLR_MAX_ occurs at the second step of the degradation.

**Table 5:** Thermogravimetric data for TPU/salen formulation in pyrolytic conditions at 10 °C/min.

Sample	s1					Sample	s2				
	*T*_5%_ (°C)	*T*_MAX1_ (°C)	*T*_MAX2_ (°C)	*MLR* (%/°C)	*Res* (%)		*T*_5%_ (°C)	*T*_MAX1_ (°C)	*T*_MAX_ (°C)	*MLR* (%/°C)	*Res* (%)
Neat TPU	303	303	406 ^2^	1.4	5	Neat TPU	303	354	406 ^2^	1.4	5
**s1**	287	347	391 ^2^	1.2	7	**s2**	298	344	407 ^2^	1.2	9
**s1-Cu**	301	343	396 ^2^	1.1	9	**s2-Cu**	303	324	377 ^2^	2	11
**s1-Mn**	N/A	N/A	N/A	N/A	N/A	**s2-Mn**	300	323	376 ^2^	1.4	11
**s1-Ni**	300	306	363	2.7 ^2^	10	**s2-Ni**	308	327	386 ^2^	1.4	7
**s1-Zn**	297	323	-	2.9 ^1^	15	**s2-Zn**	306	309	366 ^2^	1.4	15

^1^ MLR_Max_ occurs at the first step of the degradation; ^2^ MLR_MAX_ occurs at the second step of the degradation.

**Table 6:** Summary of the peak heat release rate, temperature at peak heat release rate and total heat released for the TPU/salen (90/10) formulations. pHRR: peak heat release rate; THR: total heat released.

Sample	pHRR (W/g)	*T* (°C) @ pHRR (W/g)	THR (kJ/g)
Neat TPU	1535	435	90
TPU + **s1**	2659 (+73%)	431(−4 °C)	116 (+29%)
TPU + **s1-Cu**	763 (−50%)	431(−4 °C)	52 (−42%)
TPU + **s1-Ni**	1910 (+24%)	414 (−21 °C)	65 (−28%)
TPU + **s1-Zn**	594 (−61%)	346 (−89 °C)	33 (−63%)
TPU + **s2**	608 (−60%)	430 (−5 °C)	54 (−40%)
TPU + **s2-Cu**	680 (−56%)	417 (−18 °C)	42 (−53%)
TPU + **s2-Mn**	756 (−51%)	402 (−33 °C)	38 (−58%)
TPU + **s2-Ni**	808 (−47%)	420 (−15 °C)	39 (−57%)
TPU + **s2-Zn**	1246 (−19%)	411 (−24 °C)	46 (−49%)

**Table 7:** Summary of the results for the MLC test (50 × 50 × 3 mm^3^).

Sample	pHRR (kW/m^2^)	THR (MJ/m^2^)
Neat TPU	277	66.7
TPU + **s1**	260 (−6%)	65.5 (−2%)
TPU + **s1-Cu**	244 (−11%)	48.5 (−27%)
TPU + **s1-Ni**	256 (−7%)	51.9 (−22%)
TPU + **s1-Zn**	420 (+51%)	54.3 (−18%)
TPU + **s2**	200 (−27%)	50.5 (−24%)
TPU + **s2-Cu**	187 (−32%)	45.0 (−32%)
TPU + **s2-Mn**	192 (−30%)	47.3 (−29%)
TPU + **s2-Ni**	251 (−9%)	54.9 (−17%)
TPU + **s2-Zn**	326 (+17%)	52.4 (−21%)
